# A data base of contributions of major oceanic and terrestrial moisture sources on continental daily extreme precipitation

**DOI:** 10.1016/j.dib.2021.106830

**Published:** 2021-02-03

**Authors:** Marta Vázquez, Raquel Nieto, Margarida L.R. Liberato, Luis Gimeno

**Affiliations:** a Environmental Physics Laboratory (EPhysLab), CIM-UVIGO, Universidade de Vigo, 32004 Ourense, Spain; b Instituto Dom Luiz, Universidade de Lisboa, 1749-016 Lisboa, Portugal; c Escola de Ciências e Tecnologia, Universidade de Trás-os-Montes e Alto Douro, Vila Real, Portugal

**Keywords:** Extreme precipitation, Moisture transport, Moisture sources, Lagrangian Modelling

## Abstract

Most of the moisture transported in the globe has its origin in the well-known main moisture sources defined by Gimeno et al. [1]. They provide moisture for precipitation over continental areas in the world in different proportions. This paper presents the daily moisture contribution over each 0.5 × 0.5 continental gridded point from the three preferred moisture sources (primary, secondary, and tertiary) for continental extreme precipitation during the Peak Precipitation Month. The data consist of the moisture contribution (|E−P<0|) field by month from the three preferred sources obtained using an approach based on the Lagrangian particle dispersion model FLEXPART. The data here presented is directly related to the results presented in Vazquez et al. [2].

**Specifications Table**

**Table:** 

Subject	Climatology
Specific subject area	Extreme precipitation, moisture transport, climate change
Type of data	NetCDF data files
How data were acquired	Post-processing of the FLEXPART Lagrangian particle dispersion model outputs
data format	analysed
Parameters for data collection	3D (time, longitude, latitude)) (|E−P<0|) data with 0.5 °x0.5 ° horizontal resolution achieved daily for the period 1980–2018 considering at every grid point only the Peak Precipitation Month.
Description of data collection	The data was obtained by post-processing FLEXPART model global outputs.
Data source location	Institution: University of Vigo City/Town/Region: Ourense Country: Spain Primary data sources: ECMWF ERA-Interim reanalysis data (https://www.ecmwf.int/en/forecasts/datasets/reanalysis-datasets/era-interim)
Data accessibility	With the article and in http://dx.doi.org/10.17632/kgvsvx77h8.1
Related research article	Vázquez M., Nieto R., Liberato M.L.R., Gimeno L. (2020) Atmospheric moisture sources associated with extreme precipitation during the peak precipitation month. Weather and Climate Extremes, 30, 100,289. https://doi.org/10.1016/j.wace.2020.100289

## Value of the Data

• The data synthesize the moisture contribution transported from the main global moisture sources towards continents in the month of the highest mean precipitation.
• The data results relevant to extreme precipitation studies to analyse the moisture transport effect and contribution of main global moisture sources over continetal areas.
• This data can be reused for statistical studies on moisture transport such as trend analysis

## 1 Data Description

**Fig. 1:**
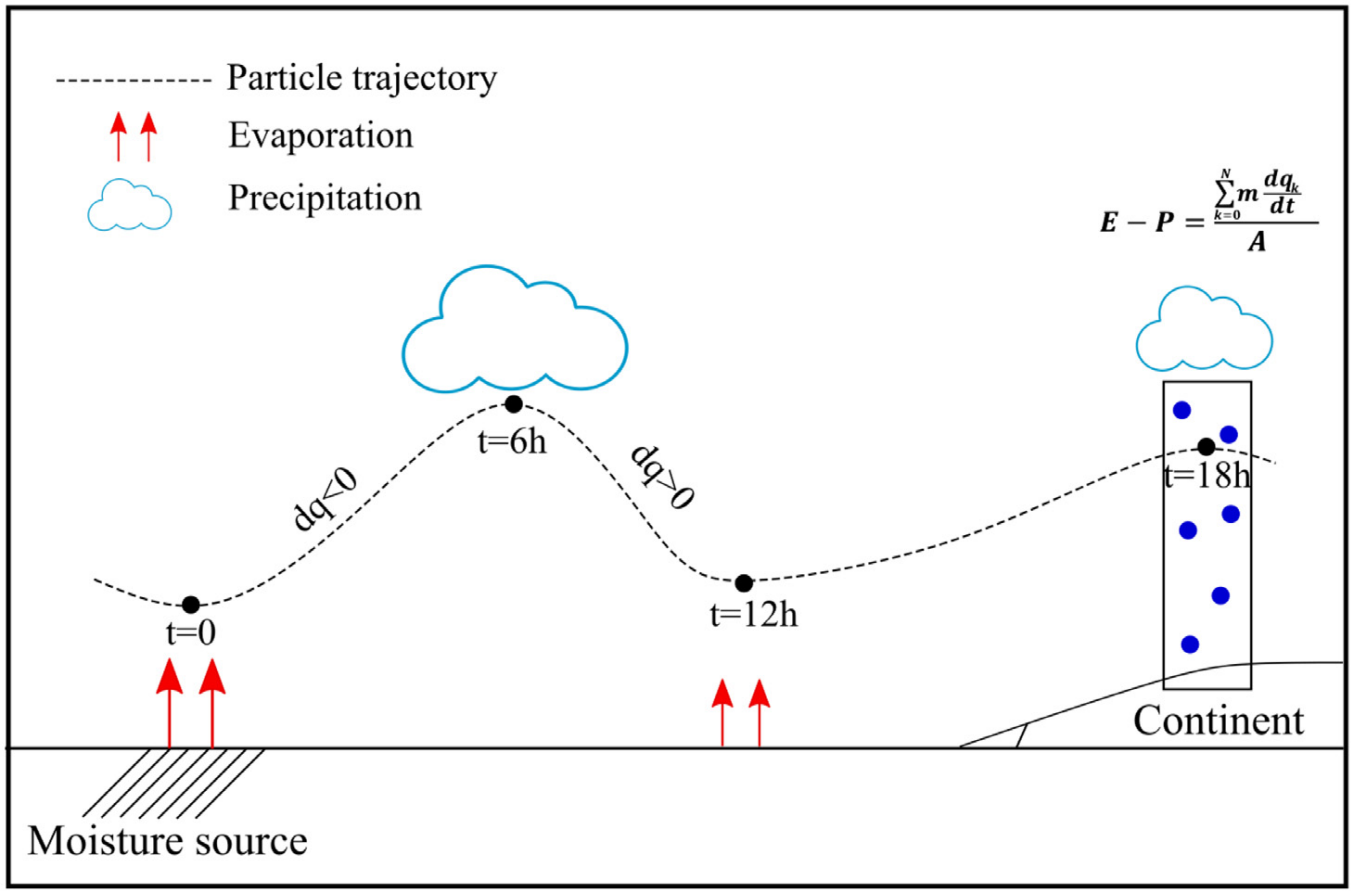
Schematic representation of moisture contribution computation.

The data presents the total moisture transport for precipitation (measured as |(E−P)<0| – being E−P evaporation minus precipitation-, in mm/day) from the Preferred, Secondary, and Tertiary Sources associated exclusively with the daily extreme precipitation events (hereinafter referred to as PS, SS, and TS, respectively) towards each grid cell of continental areas during the Peak Precipitation Month (PPM). The PPM over each grid point is defined as the month with the highest mean precipitation computed for the period 1980–2018 (as described in Nieto et al. [3]). PS, SS and TS are defined following the methodology stablish by Nieto et al. [3] where PS is defined as those source showing the highest monthly moisture contribution over each individual continental grid point. The global main oceanic and continental moisture sources are listed in Table 1, and defined in Vazquez et al. [2]. The SS and TS are those providing the second and the third highest monthly moisture contribution. For PS, SS, and TS definition, only the contribution during the extreme precipitation days is considered; being these days defined as those showing precipitation higher than the 95th percentile. Both, PPM and sources, were previously individually computed for every grid point by Vázquez et al. [2] (see Fig. 1 and Figure S1 in Vázquez et al. [2]).

**Table 1:** The main moisture source regions and the associated code (MC).

Moisture source region	Code
Agulhas Current (AGU)	1
Coral Sea (CORALS)	2
Indian Ocean (IND)	3
Mediterranean Sea (MED)	4
Gulf of Mexico and Caribbean Sea (MEXCAR)	5
North Atlantic Ocean (NATL)	6
Red Sea (REDS)	7
Southern Africa (SAFR)	8
Sahel region (SAHEL)	9
South America (SAM)	10
South Atlantic Ocean (SATL)	11
South Pacific Ocean (SPAC)	12
Zanzibar Current and Arabian Sea (ZANAR)	13
North Pacific Ocean (NPAC)	14

The data are presented as a set of NetCDF data files. In total, three files are presented, one for each source level: the PS, SS, and TS. For all of them, the spatial information is provided in a regular grid in longitude (LON) and latitude (LAT) with a 0.5 ° horizontal resolution (LAT × LON = 320 × 760 grid points). The data are presented in a daily temporal scale (T), the days in the PPM that ranges from 1 to 1209, the 31 days’ maximum month length for the 39 years (1980–2018) considered in the study. So, the final resolution of the dataset is 1209 × 360 × 720. As the datasets synthesize the information from the PS, SS, and TS and they are associated only with the PPM, it should be used combined with the dataset provided as supplementary data material and defined and explained in Vázquez et al. [2]. It is important to notice that the PPM varies geographically, as well as the associated PS, SS, and TS, as they are individually computed for each individual grid point. As not all the months have the same length, some grid points in the data files present the complete time series of 31 days, while the other 28 or 30 days, being the remaining data values completed as NaN. Leap days are not considered in this data.

To complete the information, the supplementary data files present four additional NetCDF files (PPM.nc, PS.nc, SS.nc, and TS.nc). The supplemetary data can be downloas from the Mendeley Data repository [4]. One of them presents the continental PPM for each grid point (PPM.nc), where the PPM is the month showing the highest mean precipitation, previously computed in Vázquez et al. [2] using monthly mean values from the daily precipitation data from CPC Global Unified Gauge-Based Analysis of Daily Precipitation [5]. In this file, the months from January to December are numbered from 1 to 12, and as the original data, the horizontal resolution is 0.5 ° resulting in a 360 × 720 data size. The other three NetCDF files present the PS, SS, and TS, respectively, with the same horizontal resolution. A numerical code (MC), see Table 1, is associated with each main moisture source analysed. The sources from this list were ranked according to their percentage in providing moisture for precipitation over each grid point. So, the source that provides the highest mean moisture contribution (in extreme precipitation days during the PPM is defined as the PS. Likewise, the second and third highest contribution is associated with SS and TS. To obtain these three Supplementary files (PS.nc, SS.nc, and TS.nc), at every gridded area the MC representing the PS (and SS or TS when adequate) is retained and associated with that specific points. So, in the final files, PS, SS, and TS are represented by the numerical codes MCs (ranging from 1 to 14) listed in Table 1. The resulting PS, SS, and TS can be observed in Figures S1 on Vazquez et al. [2].

## 2 Experimental Design, Materials and Methods

The data here presented is obtained by using the Lagrangian Particle Dispersion Model FLEXPART v9.0 [6], [7], [8]. This model allows following the trajectory of particles backward or forward in time in order to analyse the moisture changes experienced by them. The model uses the reanalysis data from the European Centre for Medium-Range Weather Forecasts (ECMWF), specifically the ERA-Interim data product [9]. The variables needed to feed up the model are listed in Table 2. This data was downloaded at 1 ° horizontal resolutions at 61 vertical levels and with a 6 h time step (more details in Vazquez et al. [2]) for a long period from 1980 to 2018, using flex_extract open-source software [10]

**Table 2:** Input ERA-Interim data needed for FLEXPART experiment.

Bidimensional data	Three-dimensional data
Surface Pressure	Horizontal and vertical wind components
Total cloud cover	Temperature
10 m horizontal wind components	Specific humidity
2 m and dew point temperature	
Large scale and convective precipitation	
Sensible heat flux	
East/west and north/south surface stress	
Topography and subgrid standard deviation	

ERA-Interim to describe the movement of the particles initially released and it provides, every 6 h, the position and the specific humidity (as well as other variables listed in Table 3) of each particle along their trajectories in the experiment. From these outputs, the methodology established by Stohl and James [11], [12] can be applied in order to investigate the moisture transport. In this case, to find the sinks of moisture, the particles were followed forward in time. From the global experiment outputs, the particles which leave every main global source of moisture (those listed in Table 1) every day at every 6 h are selected and their characteristics along each trajectory during 15 days (the time considered enough to take into account the residence time of water vapour in the atmosphere [13]) were retained for our final purpose. So, for each particle the moisture variation (e−p) was computed as $e-p=m\frac{dq}{dt}$, where m is the mass of the particle, dq is the variation in the specific humidity between two time steps, and dt the time step (dt=6 h). Once the individual trajectories for all the particles were computed, the total surface freshwater flux at each grid cell can be computed by adding the contribution of all the particles that cross a specific grid area (A) at a specific time. The total budget is computed as expressed in Eq. (1).

(1) $$E-P=\frac{\sum_{k=1}^{N}{\left(e-p\right)}_{k}}{A}$$

**Table 3:** FLEXPART output variables.

Variable	Units
Latitude	degrees
Longitude	degrees
Height	m
Topographic height	m
Potential vorticity	10–6(m^2^Ks^−1^kg^−1^)
Specific humidity	gkg^−1^
Air density	kgm^−3^
ABL height	m
Temperature	K

In this equation, E represents evaporation, P precipitation, N is the total number of particles over the grid area, and A is the area of the grid cell where the total budget is calculated. A representation of this procedure is illustrated in Fig. 1.

As previously commented, the particles’ tracking was performed for 15 days, but it is known that the residence time of water vapour on the atmosphere varies geographically. For this reason, the computation over each continental grid point was done taking into consideration the optimal time of integration for Lagrangian approaches defined by Nieto and Gimeno [13]. So, grid to grid, the monthly optimal time of integration is used for computing E−P values. Taking this into consideration, as an example if this optimal time for a grid point is 10 days, the total E−P in a specific day is computed taking into consideration the particles that left the source between 1 and 10 days before.

It is important to notice that E−P represents the balance between evaporation and precipitation. Areas with E−P<0 represent net loss of moisture considering all the particles, on the other hand, E−P>0 regions represent net moisture uptake. Following the selected trajectories over a specific area from the FLEXPART outputs in a forward mode, results in the moisture contribution for precipitation from it over other regions. For this reason, once the daily E−P were computed at each gridded point, the positive values (E−P>0) were removed, and the |E−P<0| field is retained and presented in the final dataset.

For every main global source in Table 1 the moisture contribution for precipitation (|E−P<0|) was obtained daily following this procedure for every month and the complete period 1980–2018. To synthesize the information, at each gridded area, the PPM is selected according to the supplementary data (PPM.nc) included in Supplementary Material, and only the information from that month was retained. After that, a total of 14 fields of |E−P<0| are obtained, representing the moisture contribution for precipitation during the PPM for each main moisture source.

On the next step, the PS, SS, and TS are selected grid to grid according to the Supplementary Files PS.nc, SS.nc, and TS.nc, respectively. From the 14 initial fields of |E−P<0| a single file is constructed for each of the three sources’ level (PS, SS, and TS), in which only the information from the source that acts as PS, SS, and TS (respectively for each of the files here presented) is included in the files named as EP_PS.nc, EP_SS.nc and EP_TS.nc.

## CRediT Author Statement

**Marta Vázquez:** Conceptualization, Methodology, Visualization, Investigation, Writing – original draft Writing– review & editing; **Raquel Nieto:** Conceptualization, Methodology, Supervision, Writing – review & editing; **Margarida L.R. Liberato:** Methodology, Supervision, Writing – review & editing; **Luis Gimeno:** Conceptualization, Supervision, Writing – review & editing.

## Declaration of Competing Interest

The authors declare that they have no known competing financial interests or personal relationships which have or could be perceived to have influenced the work reported in this article.
